# Patterns of Failure in Synchronous Metastatic Non-Small Cell Lung Cancer Without Driver Alterations According to Metastatic Burden

**DOI:** 10.3390/cancers18091363

**Published:** 2026-04-24

**Authors:** Woo Joong Rhee, Sangjoon Park, Jee Suk Chang, Hong In Yoon, Jaeho Cho, Kyung Hwan Kim

**Affiliations:** Department of Radiation Oncology, Yonsei Cancer Center, Heavy Ion Therapy Research Institute, Yonsei University College of Medicine, 50-1 Yonsei-ro, Seodaemun-gu, Seoul 03722, Republic of Korea; wjrhee@yuhs.ac (W.J.R.); jjhmd@yuhs.ac (J.C.)

**Keywords:** synchronous metastatic non-small cell lung cancer, driver-negative, patterns of failure, number of metastatic lesions

## Abstract

Background: Patterns of failure (POFs) after first-line immune checkpoint inhibitor (ICI)-based therapy in patients with synchronous metastatic non-small cell lung cancer (NSCLC) without oncogenic driver alterations may guide the selection of candidates for local consolidative therapy (LCT). Methods: We retrospectively evaluated patients diagnosed with synchronous metastatic NSCLC between January 2017 and December 2023. Patients with oncogenic driver alterations, those who did not receive ICIs as first-line therapy, or those who lacked follow-up imaging were excluded. Patients were stratified into four groups according to the number of metastatic lesions: 1, 2, 3–5, and >5 lesions. POFs were classified as original site recurrence (OSR) or new site recurrence with or without OSR (NSR). Competing risk analyses were performed. Results: A total of 221 patients were analyzed, with a median follow-up of 28.1 months. Initial failure patterns did not differ significantly across lesion-number groups (*p* = 0.417). The 2-year cumulative incidence of OSR was not significantly different between the groups (*p* = 0.828). A trend toward a lower NSR was observed in patients with a single metastatic lesion (*p* = 0.063). Analysis of subsequent failures revealed a higher rate of NSR in the 1-lesion group than in the other groups (*p* = 0.043). No independent predictors of OSR were identified in multivariate analysis. Conclusions: In synchronous metastatic driver-negative NSCLC treated with first-line ICI-based therapy, both OSR and NSR were common and not clearly associated with metastatic burden, suggesting that lesion number alone may be insufficient for selecting candidates for LCT.

## Simple Summary

Determining optimal candidates for local treatment in metastatic non-small cell lung cancer remains a significant clinical challenge. In this study, we investigated patterns of failure after first-line immune checkpoint inhibitor-based therapy in patients with synchronous metastatic non-small cell lung cancer without oncogenic driver alterations. We assessed whether the number of metastatic lesions predicts the site of recurrence, specifically original site recurrence and new site recurrence. Our findings revealed that both types of recurrence were common and not clearly associated with metastatic burden. These results suggest that metastatic burden alone may be an insufficient criterion for selecting candidates for local treatment. More comprehensive approaches incorporating factors beyond metastatic burden are needed to improve patient selection and optimize treatment strategies.

## 1. Introduction

Historically, the treatment paradigm for patients with metastatic cancer has predominantly relied on systemic therapies, with local modalities such as radiotherapy or surgery largely reserved for palliative purposes [1,2,3]. However, the concept of the oligometastatic state, an intermediate stage between localized and widespread disease, has led to increasing interest in aggressive local consolidative therapy (LCT) [4]. Randomized trials across various malignancies, such as the SABR-COMET trial, have demonstrated that the addition of stereotactic ablative radiotherapy (SABR) to the standard of care significantly improves overall survival (OS) in patients with limited metastatic burden [5].

In non-small cell lung cancer (NSCLC), several studies, including those by Gomez et al. and Iyengar et al., have demonstrated significant improvements in progression-free survival (PFS) and OS with the addition of LCT in patients who achieved a treatment response or stable disease after systemic therapy [6,7]. The CURB trial also evaluated the efficacy of SABR in patients with metastatic NSCLC or breast cancer who developed oligoprogression after systemic therapy. Although no PFS benefit was observed in patients with breast cancer, SABR significantly prolonged PFS in the NSCLC cohort [8]. Moreover, in patients with epidermal growth factor receptor (EGFR)-mutated NSCLC treated with tyrosine kinase inhibitors (TKIs), upfront radiotherapy was associated with significantly improved survival, as demonstrated in the SINDAS trial [9]. These findings suggest that LCT is a promising strategy for selected patients with oligometastatic disease.

However, the role of LCT in the era of contemporary immune checkpoint inhibitors (ICI) remains unclear. The NRG-LU002 trial, a randomized phase II/III study, evaluated the benefit of LCT in patients with NSCLC without driver alterations who had three or fewer extracranial metastatic lesions and achieved at least stable disease after four cycles of first-line systemic therapy. Notably, approximately 90% of patients received ICI-based therapy. In both the overall population and the ICI-treated subgroup, the addition of LCT did not result in significant improvements in PFS or OS [10]. These conflicting results have introduced uncertainty into clinical decision-making regarding patient selection for aggressive local therapy, particularly in the context of immunotherapy-based first-line regimens for driver-negative NSCLC.

Although many previous studies have focused on patients who achieved at least stable disease after systemic therapy or those with metachronous oligometastatic disease, high-level evidence regarding the efficacy of LCT in synchronous oligometastatic disease remains limited. Consequently, the role of LCT in this clinical setting remains uncertain. The SABR-SYNC trial is currently underway to address this question [11]. Given that approximately 40–50% of patients with NSCLC present with stage IV disease at initial diagnosis, identifying patients with synchronous oligometastatic disease who may benefit from local therapy remains a clinically important challenge [12,13,14]. Therefore, the present study aimed to analyze the patterns of failure (POFs) after first-line ICI-based systemic therapy in patients with de novo metastatic NSCLC without driver alterations. Furthermore, we sought to identify predictive factors for oligoprogression or original site-confined recurrence to define patient subgroups that may benefit from the addition of local therapy.

## 2. Materials and Methods

### 2.1. Patients

**Figure 1:**
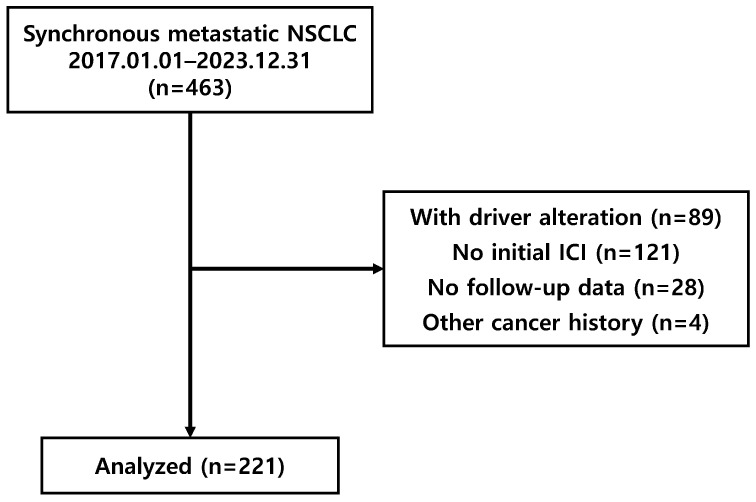
Study flow diagram of patient selection. A total of 463 patients were screened, and 221 patients with synchronous metastatic NSCLC without driver alterations who were treated with first-line ICI-based therapy were included in the analysis.

We retrospectively collected data from patients with histologically confirmed synchronous metastatic NSCLC who were treated with PD-1 and PD-L1 inhibitors at the Yonsei Cancer Center between January 2017 and December 2023. Consecutive patients were identified using an institutional patient search system with predefined keywords (“NSCLC,” “metastasis,” and “ICI use”), and all eligible patients identified through this process were screened and included. Patients were excluded if they had oncogenic driver alterations (e.g., *EGFR*, *ALK*, or *ROS1*), did not receive ICIs as first-line therapy, lacked follow-up imaging studies, or had a history of another malignancy within five years prior to NSCLC diagnosis. A study flow diagram is shown in Figure 1. Data on clinical characteristics, including age, sex, histology, clinical TNM classification, primary tumor size, PD-L1 expression level, number of metastatic lesions and organs, systemic treatment scheme, and ICI regimen were collected. This study was approved by the Institutional Review Board of Severance Hospital (IRB No. 4-2024-1321). Informed consent was waived because of the retrospective nature of the study.

At the time of diagnosis, all patients underwent chest computed tomography (CT), abdomino-pelvic CT (AP-CT), positron emission tomography–computed tomography (PET-CT), and brain magnetic resonance imaging (MRI) to assess disease extent. Follow-up imaging after treatment included chest CT and AP-CT every 3 months. PET-CT scans were performed if abnormal findings were detected. Patients with brain metastases at diagnosis underwent follow-up brain MRI every 3–6 months. Patients with squamous cell carcinoma were primarily treated with pembrolizumab in combination with carboplatin and paclitaxel, whereas those with nonsquamous histology were primarily treated with pembrolizumab, carboplatin, and pemetrexed. Pembrolizumab monotherapy was administered to selected patients with a PD-L1 TPS ≥ 50%. Local treatments for brain metastases included gamma knife surgery (GKS) or whole-brain radiotherapy, and palliative radiotherapy was delivered to symptomatic lesions. LCT was not performed owing to the institutional treatment policy.

### 2.2. Patterns of Failure Analysis

Metastatic lesions were defined based on a comprehensive review of all available imaging studies, including CT, MRI, and PET/CT, performed at the time of diagnosis. All imaging studies were systematically reviewed to identify metastatic lesions, excluding primary tumors and regional lymph node (LN) metastases. Imaging assessments were conducted by multiple board-certified radiation oncologists who are coauthors of this study. The patients were categorized into four subgroups according to the number of metastatic lesions: 1, 2, 3–5, and >5 lesions. Metastatic lesions were counted based on radiographically distinguishable discrete lesions. Patients with malignant pleural effusion, pericardial effusion, pleural seeding, leptomeningeal seeding, or peritoneal carcinomatosis were classified into the >5 metastases group, as these patterns represent diffuse disease dissemination for which accurate and reproducible lesion-by-lesion quantification is not feasible. Conglomerated nonregional LN metastases within the same nodal station were counted as a single metastatic lesion.

Disease recurrence was assessed based on the clinical presentation and follow-up imaging findings. Failure sites were classified as either original sites, defined as recurrences within the initially involved disease sites, or new sites, defined as recurrences outside the initial disease extent. For POF analysis, recurrence was further categorized into two groups: original site recurrence (OSR) (defined as failure occurring exclusively at the original sites) and new site with or without original site recurrence (NSR) (defined as recurrence involving new sites, regardless of the presence of OSR). Recurrence at previously irradiated or locally treated lesions was also classified as OSR. Recurrence within the original sites was further classified as locoregional or metastatic failure. In addition, subsequent POFs were analyzed to determine whether disease progression met the criteria for limited progression. Limited progression was defined as disease progression involving five or fewer lesions.

### 2.3. Statistical Analysis

Chi-square or Fisher’s exact tests were used to compare categorical variables, and the Mann–Whitney U test was used to compare continuous variables. The cumulative incidence of recurrence was defined as the time from treatment initiation to the occurrence of OSR or NSR and was estimated and compared using competing risk analysis with the Fine–Gray model. In the competing risk analysis, competing events included alternative recurrence patterns (e.g., NSR as a competing event for OSR and vice versa) and death without recurrence. Fine–Gray regression analyses were performed for both univariable and multivariable models, with backward stepwise selection applied for multivariable model selection. OS was defined as the time from treatment initiation to death from any cause, whereas PFS was defined as the time from treatment initiation to disease progression or death. OS and PFS were estimated using the Kaplan–Meier method and compared using the log-rank test. A two-sided *p* value of less than 0.05 was considered statistically significant. Statistical analyses were performed using the SPSS, version 28.0.0.0 (IBM Corp., Armonk, NY, USA) and R, version 4.4.3 (R Foundation for Statistical Computing, Vienna, Austria).

## 3. Results

### 3.1. Patient Characteristics

A total of 463 patients were screened, and 221 were analyzed (Figure 1). The median follow-up duration was 28.1 months (range, 2.7–103.6 months). The patients were categorized into four subgroups according to the number of metastatic lesions at diagnosis: one lesion (*n* = 28), two lesions (*n* = 24), three–five lesions (*n* = 20), and more than five lesions (*n* = 149).

The baseline clinical characteristics are summarized in Table 1. Most variables, including age, sex, histology, N classification, and PD-L1 expression levels, were comparable among the four groups. However, patients with a higher metastatic burden were more likely to have an advanced clinical T stage (*p* < 0.001) and larger primary tumor size (*p* = 0.045). Locations of the metastatic lesions are summarized in Supplementary Table S1. There were no significant differences in the systemic treatment schemes or ICI regimens among the subgroups (Supplementary Table S2). Overall, 11 patients underwent GKS and 22 patients received palliative radiotherapy.

### 3.2. Patterns of First Failure

**Figure 2:**
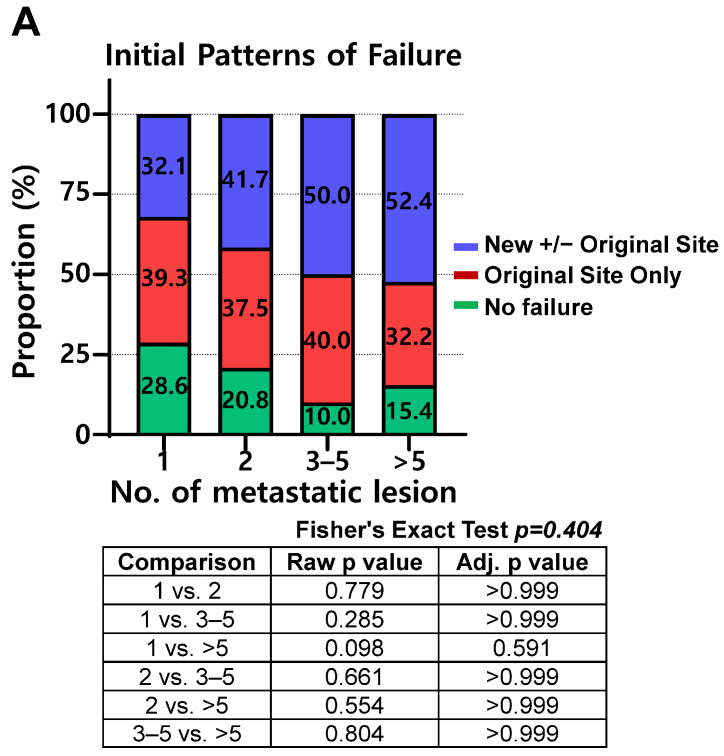

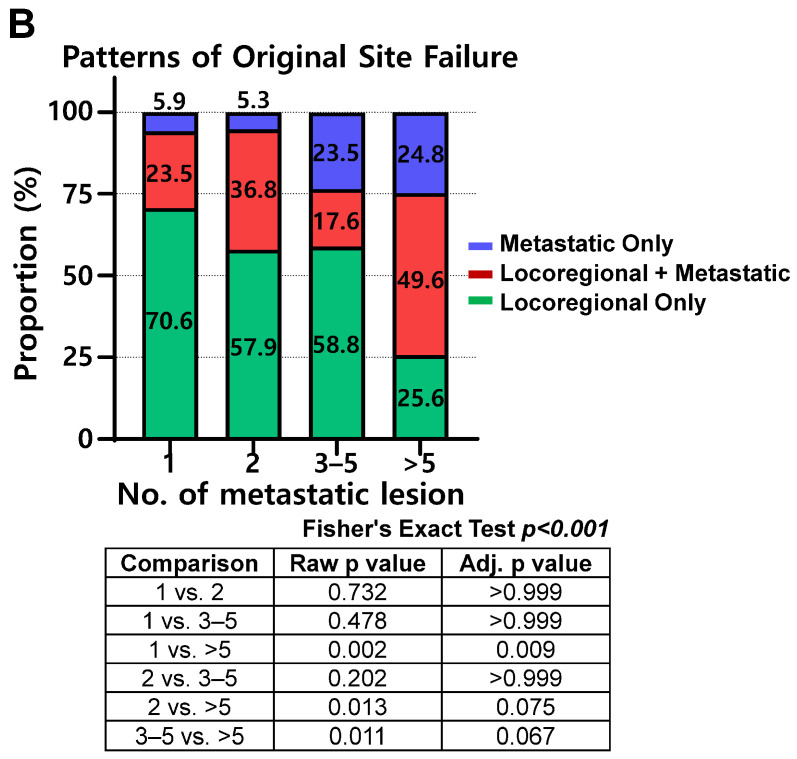
Patterns of failure after first-line treatment according to number of metastatic lesions. (**A**) Distribution of patterns of failure across four subgroups (1, 2, 3–5, and >5 lesions). (**B**) Distribution of patterns of failure within original site recurrence across four subgroups (1, 2, 3–5, and >5 lesions), classified as locoregional-only failure, metastatic-only failure, or combined locoregional and metastatic failure.

Initial POFs after first-line therapy were analyzed according to metastatic burden. The proportion of OSR did not differ significantly among the four subgroups (Figure 2A). However, a detailed analysis of OSR revealed that with increasing metastatic burden, the proportion of locoregional-only failures decreased, whereas the proportion of metastatic failures with or without locoregional involvement increased (Figure 2B). Among the 183 patients who experienced disease progression, the rates of limited progression were 55.0%, 47.4%, 61.6%, and 25.4% in the 1-, 2-, 3–5-, and >5-lesion groups, respectively (*p* = 0.002).

**Figure 3:**
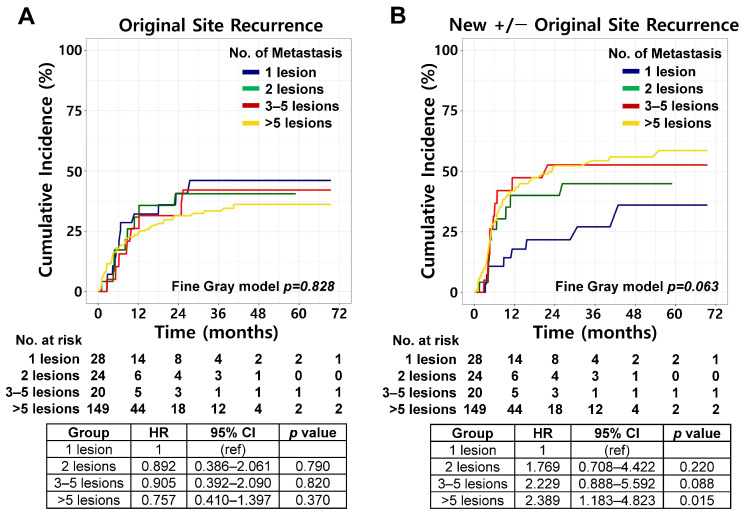

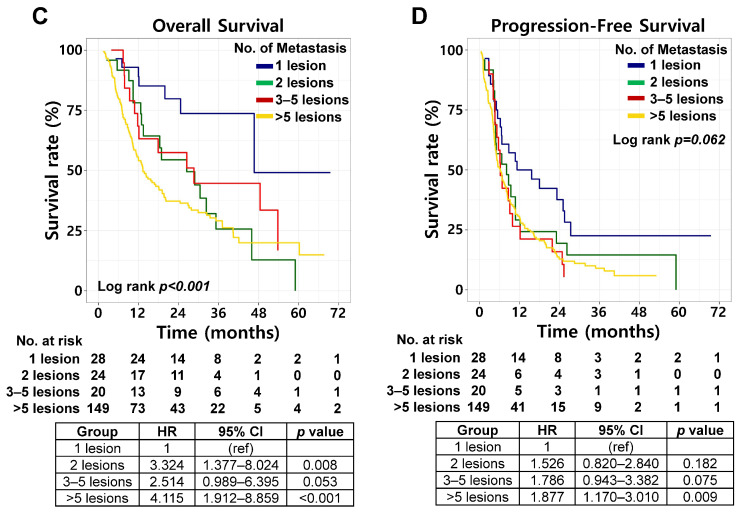
Clinical outcomes according to number of metastatic lesions. (**A**) Cumulative incidence of original site recurrence according to number of metastatic lesions, estimated using competing risk analysis. (**B**) Cumulative incidence of new site recurrence with or without original site recurrence according to number of metastatic lesions, estimated using competing risk analysis. (**C**) Overall survival stratified by number of metastatic lesions. (**D**) Progression-free survival stratified by number of metastatic lesions.

Competing risk analysis demonstrated no significant differences in the 2-year cumulative incidence of OSR among the four groups: 40.7% (95% CI, 25.2–65.6%), 40.5% (95% CI, 23.7–69.3%), 31.5% (95% CI, 15.5–63.8%), and 31.4% (95% CI, 24.5–40.3%) for 1-, 2-, 3–5-, and >5-lesion groups, respectively (Fine–Gray *p* = 0.828; Figure 3A). However, a trend toward a lower incidence of NSR was observed in patients with a single metastatic lesion compared with those with higher metastatic burden: 21.7% (95% CI, 10.5–45.0%), 40.1% (95% CI, 23.6–68.0%), 52.6% (95% CI, 33.4–83.0%), and 52.3% (95% CI, 44.5–61.5%) for 1-, 2-, 3–5-, and >5-lesion groups, respectively (Fine–Gray *p* = 0.063; Figure 3B). Additional subgroup analyses stratified by PD-L1 expression level (≥50% vs. <50%), systemic treatment regimen (ICI monotherapy vs. combination), and histology (SCC vs. Non-SCC) were performed (Supplementary Figure S1A–L). Consistent with the findings in the overall cohort, the cumulative incidence of OSR did not differ significantly according to metastatic burden in any subgroup (all Fine-Gray *p* > 0.05). For NSR, a higher metastatic burden remained significantly associated with an increased cumulative incidence in selected subgroups, including patients with non-squamous histology, PD-L1 < 50%, and those receiving ICI monotherapy. In contrast, no significant associations were observed in the SCC, PD-L1 ≥ 50%, or ICI combination subgroups.

The effect of baseline metastatic sites on the patterns of first failure was further evaluated (Supplementary Table S3). In the overall cohort, patients with bone metastases at diagnosis exhibited a significantly higher NSR rate (60.8% vs. 41.5%) and a lower rate of no failure (8.9% vs. 21.8%) than did those without bone metastases (*p* = 0.009). This pattern was not observed in the 1-, 2-, or 3–5-lesion groups but was evident only in the >5 metastatic lesion group. Baseline brain or liver involvement was not significantly associated with the first failure pattern.

Univariable and multivariable analyses were performed to identify predictors of original site-confined recurrence (Table 2). On univariable analysis, older age (HR 1.028, 95% CI, 1.001–1.056, *p* = 0.039), nonsquamous histology (HR 0.602, 95% CI, 0.371–0.977, *p* = 0.040), and involvement of two metastatic organs (HR 0.489, 95% CI, 0.266–0.901, *p* = 0.022) were associated with OSR. However, none of these factors remained significant in multivariable analysis.

OS differed significantly among the four groups (log-rank *p* < 0.001; Figure 3C). The 2-year OS rates were 79.8% (95% CI, 57.5–91.2%), 54.4% (95% CI, 31.7–72.5%), 57.4% (95% CI, 32.5–76.0%), and 37.2% (95% CI, 29.2–45.3%) in the 1-, 2-, 3–5-, and >5-lesion groups, respectively. PFS showed a trend toward worse outcomes with an increased metastatic burden (log-rank *p* = 0.062; Figure 3D). The corresponding 2-year PFS rates were 37.6% (95% CI, 19.8–55.4%), 19.4% (95% CI, 6.2–38.0%), 15.9% (95% CI, 3.9–35.1%), and 12.8% (95% CI, 7.9–19.0%), respectively. Independent of baseline metastatic burden, survival outcomes were significantly associated with the pattern of progression after first-line therapy. Patients who experienced oligoprogression demonstrated significantly superior OS compared to those with polyprogression, with 2-year OS rates of 66.3% (95% CI, 52.7–76.8%) versus 26.9% (95% CI, 19.0–35.5%), respectively (log-rank *p* < 0.001; Supplementary Figure S2A). Similarly, PFS was significantly better in the oligoprogression group, with 2-year rates of 9.5% (95% CI, 3.9–18.2%) compared with 5.0% (95% CI, 2.1–9.9%) in the polyprogression group (log-rank *p* = 0.040; Supplementary Figure S2B).

### 3.3. Patterns of Subsequent Failure

Patients who experienced disease progression after first-line therapy received supportive care, systemic therapy, local salvage therapy, or combined systemic and local salvage therapy (Table 3). Chemotherapy alone was the most frequently administered second-line systemic therapy. The time to subsequent failure after second-line therapy did not differ across the patient groups. In the patterns of subsequent failure analyses, new sites with or without previous site involvement were most frequent in the 1-lesion group, occurring in 46.4% of the patients. In addition, the rate of limited progressive disease at subsequent failure was lowest in the 1-lesion group with a trend toward statistical significance (*p* = 0.057).

Among patients who developed disease progression after first-line therapy, subsequent patterns of failure were analyzed in 20 patients who underwent local salvage therapy (Supplementary Table S4). Of these, 18 received radiotherapy, 1 underwent GKS, and 1 received both radiotherapy and GKS. Patients who received local salvage therapy demonstrated significantly improved OS compared with those who did not (2-year OS rate 69.1% [95% CI, 43.6–84.8%] vs. 37.1% [95% CI, 29.3–44.8%]; log-rank *p* = 0.007; Supplementary Figure S3A). In contrast, PFS did not differ significantly between the two groups (2-year PFS rate 15.0% [95% CI, 3.7–33.5%] vs. 5.5% [95% CI, 2.7–9.8%] in the local salvage and no local salvage groups, respectively; log-rank *p* = 0.131; Supplementary Figure S3B). The proportion of patients with limited progression at initial failure was significantly higher in those who received local salvage therapy than in those who did not (80.0% vs. 28.8%, *p* < 0.001). In the analysis of subsequent patterns of failure, patients who received local salvage therapy exhibited a higher proportion of new site with or without previous site failure compared with those who did not (65.0% vs. 33.7%; Supplementary Table S4).

## 4. Discussion

This study identified POFs in patients with synchronous metastatic NSCLC without driver alterations who were treated with first-line ICI-containing systemic therapy. We found that the ratio of original site failure to new site failure did not differ significantly among patients with different metastatic burdens. Although multiple landmark clinical trials have demonstrated that LCT reduces original site failure and improves survival in selected patients with oligometastatic NSCLC [5,6,7], no clinical or tumor burden-related variables independently predicted OSR in our cohort. These findings emphasize the limitations of a metastatic burden-based approach for selecting patients for LCT.

Prior studies on EGFR-mutated NSCLC have shown that disease progression predominantly occurs at the original disease sites rather than at new sites [15], providing a strong biological rationale for the integration of LCT. Consistent with this rationale, several prospective trials have reported survival benefits of LCT in patients with driver-altered NSCLC [9,16,17]. By contrast, the NRG-LU002 trial, in which approximately 90% of patients received ICI-based systemic therapy, failed to demonstrate a survival advantage with the addition of LCT [10]. Subgroup analyses limited to patients treated with ICI-based regimens also showed no survival benefits, although the underlying reasons for this negative result remain unclear. A potential explanation is the relatively inferior systemic disease control achieved with ICI-based therapy compared with that achieved with targeted therapy.

In the NRG-LU002 trial, eligibility was restricted to patients with three or fewer metastatic lesions, with more than half of the enrolled patients presenting with a single metastatic lesion. Similarly, in our cohort, the cumulative incidence of OSR did not differ across lesion-number subgroups, and NSR remained frequent regardless of the metastatic burden. In addition, although patients with a single metastatic lesion showed a trend toward a lower incidence of NSR at first failure, analyses of subsequent failure patterns revealed that this group exhibited the highest rate of new lesions with or without previous site failure and the lowest rate of limited progression. These paradoxical findings may be attributed to the presence of occult micrometastatic disease in patients with a single visible metastatic lesion at diagnosis, leading to delayed systemic dissemination despite a period of systemic treatment. Therefore, until novel biomarkers beyond metastatic burden and conventional clinical parameters are validated, the integration of LCT with first-line systemic treatment in patients with synchronous metastatic NSCLC, particularly those treated with ICI-based regimens, should be approached with caution. The results of the ongoing SABR-SYNC trial will be critical for further refining patient selection [11].

In addition to the role of LCT in oligometastatic disease, increasing attention has been given to its potential role in oligoprogressive disease, where a limited number of progressing lesions emerge during systemic therapy. The CURB trial, a representative prospective study in this setting, demonstrated that local treatment for oligoprogressive disease improved PFS in patients with NSCLC, although no OS benefit was observed [8]. In addition, a recent single-institution retrospective study by Friedes et al. reported that, in patients with metastatic NSCLC treated with first-line pembrolizumab-based therapy, those with oligoprogressive disease had more favorable OS compared with those with polyprogressive disease. Furthermore, among patients with oligoprogression, radiotherapy to all progressing sites was associated with improved OS compared with a change in systemic therapy [18]. While this retrospective study included a broader metastatic NSCLC population, it suggests that local treatment may still confer clinical benefit in the context of ICI-based therapy. In our cohort, we also observed more favorable OS and PFS in patients with limited progressive disease after first-line therapy compared with those with polyprogression. These findings highlight the uncertainty regarding the clinical impact of local treatment in oligoprogressive NSCLC and underscore the need for further prospective studies to better define its role.

Further investigation is required to determine the optimal extent of local treatment strategies. In a recent phase III trial involving patients with EGFR-mutant oligo-organ metastatic NSCLC, first-line EGFR TKIs with concurrent thoracic radiotherapy targeting the primary tumor and involved regional LNs resulted in significant improvements in both PFS and OS [17]. This finding suggests that even when comprehensive treatment of all metastatic lesions is not feasible, thoracic local control may provide clinical benefits to selected patients. Although this evidence was derived from patients treated with TKIs, it remains to be determined whether a similar benefit from thoracic local control exists in patients receiving ICI-based therapy. Notably, in our study, among patients with five or fewer metastatic lesions, the majority of OSR involved locoregional thoracic disease, further supporting the potential role of thoracic local control.

Recently, several biomarkers have been investigated to predict treatment outcomes in NSCLC using ICI-based approaches. Clearance or reduction in circulating tumor DNA (ctDNA) after ICI-based therapy is associated with higher response rates and prolonged PFS and OS [19,20,21,22]. In addition, specific circulating microRNAs (miRNAs), such as miR-20a, miR-10b, miR-150, miR-223, and miR-205, have been proposed as potential predictors of response and prognosis in patients receiving ICI-based therapy [23,24,25]. Furthermore, emerging approaches integrating plasma proteome-based assays, artificial intelligence-driven radiomics, and multi-omics analyses have shown promise in refining risk stratification and predicting treatment response [26,27,28]. Although evidence remains insufficient, continuous efforts are warranted to discover biomarkers that can predict POFs and help select appropriate candidates for LCT in NSCLC.

In addition, rebiopsy at the time of disease progression may provide critical insights into tumor evolution and therapeutic resistance. Scheffler et al. reported that biomarker profiles changed in 48.9% of patients at the time of rebiopsy, with clinically relevant findings identified in 31.3% and previously unidentified targetable alterations detected in 4.4% of cases [29]. Rebiopsy-guided treatment strategies have also been associated with improved survival outcomes. For example, patients who underwent rebiopsy after osimertinib progression showed longer post-progression OS compared with those without rebiopsy (11.7 vs. 6.8 months, *p* = 0.011) [30]. In addition, dynamic changes in PD-L1 expression were observed in 33% of patients, 17% of which were clinically significant for subsequent therapy selection [31]. Therefore, when clinically feasible, rebiopsy may contribute to more refined and individualized management of advanced NSCLC.

This study has several limitations. First, the cohort was restricted to patients with synchronous metastatic NSCLC at the initial diagnosis, which may limit generalizability to metachronous metastatic disease. Nevertheless, this restriction resulted in a relatively homogeneous study population representing a clinically relevant and previously underexplored subgroup. A single-institution setting with consistent treatment policies enabled a more uniform assessment of the disease course. Second, the inclusion of only patients receiving first-line ICI-based therapy and the exclusion of those without follow-up imaging may have introduced selection bias toward patients with more favorable clinical characteristics. In addition, the absence of routine upfront LCT may have influenced the observed patterns of failure, potentially contributing to a higher rate of OSR. However, the substantial proportion of NSR suggests that systemic disease control remains a key determinant of outcomes. Third, the classification of recurrence into OSR and NSR may oversimplify the biological heterogeneity of metastatic disease, as the NSR category includes cases with or without concurrent OSR. In addition, the definition of limited progression (≤5 lesions) is not universally accepted, and classifying diffuse metastatic conditions (e.g., malignant effusions) into the >5 metastases group may have introduced prognostic heterogeneity. As the cohort lacked upfront LCT, our study is prognostic rather than predictive and does not evaluate the therapeutic efficacy of LCT. Therefore, these findings should be interpreted with caution. Fourth, only a small number of variables were retained in the final multivariable model, which may reflect limited statistical power despite efforts to minimize overfitting. Finally, modest sample sizes in the lower-burden subgroups and organ-specific analyses limit statistical power and these findings should be considered exploratory. Consequently, specific observations—such as the borderline trend toward lower NSR in the single-lesion group—should be interpreted as hypothesis-generating. Nevertheless, comprehensive analyses of subsequent failures were performed through a detailed review of follow-up imaging to enhance the accuracy of the failure classification.

## 5. Conclusions

In conclusion, among patients with synchronous metastatic, driver-negative NSCLC treated with first-line ICI-based therapy, both OSR and NSR were common and not clearly associated with metastatic burden at initial diagnosis. These findings indicate that lesion count alone is insufficient to select candidates for LCT. Future investigations should focus on identifying novel biomarkers beyond metastatic burden and conventional clinical parameters to better predict POFs, thereby enabling a more precise selection of patients who are likely to benefit from LCT.

## Figures and Tables

**Table 1:** Baseline characteristics according to number of metastatic lesions.

	1 Lesion (*N* = 28)		2 Lesions (*N* = 24)		3–5 Lesions (*N* = 20)		>5 Lesions (*N* = 149)		
	***N***	**%**	***N***	**%**	***N***	**%**	***N***	**%**	***p*** **Value**
Age (years)									0.468
Median (range)	68 (range, 54–83)		65 (range, 53–83)		67 (range, 56–86)		68 (range, 39–89)		
Sex									0.192
Male	22	78.6	20	83.3	16	80.0	120	80.5	
Female	6	21.4	4	16.7	4	20.0	29	19.5	
Histology									0.759
SCC	6	21.4	4	16.7	6	30.0	32	21.5	
Non-SCC	22	78.6	20	83.3	14	70.0	117	78.5	
Clinical T classification									<0.001
T1–T2	16	57.1	9	37.5	2	10.0	37	24.8	
T3–T4	12	42.9	15	62.5	18	90.0	112	75.2	
Clinical N classification									0.972
N0	3	10.7	2	8.3	1	5.0	10	6.7	
N1	2	7.1	3	12.5	2	10.0	13	8.7	
N2	9	32.1	9	37.5	8	40.0	45	30.2	
N3	14	50.0	10	41.7	9	45.0	81	54.4	
Clinical M classification									<0.001
M1a	5	17.9	1	4.2	3	15.0	59	39.6	
M1b	23	82.1	8	33.3	3	15.0	24	16.1	
M1c	0	0.0	15	62.5	14	70.0	66	44.3	
Primary tumor size (cm)									0.045
Median (range)	4.0 (range, 1.2–7.3)		4.35 (range, 1.5–12.5)		5.2 (range, 3.1–12.7)		4.8 (range, 1.1–13.0)		
PD-L1 expression									0.534
<1	11	39.3	10	41.7	7	35.0	44	29.5	
1–49	12	42.9	7	29.2	6	30.0	51	34.2	
≥50	5	17.9	5	20.8	7	35.0	42	28.2	
N/A	0	0.0	2	8.3	0	0.0	12	8.1	
Number of metastatic organs									<0.001
1	28	100.0	12	50.0	12	60.0	45	30.2	
2	0	0.0	12	50.0	6	30.0	35	23.5	
3	0	0.0	0	0.0	2	10.0	34	22.8	
>3	0	0.0	0	0.0	0	0.0	35	23.5	

Abbreviations: SCC, squamous cell carcinoma.

**Table 2:** Univariable and multivariable Fine–Gray regression analyses for predictors of original site recurrence.

		Univariable			Multivariable	
Variable	HR	95% CI	*p* Value	aHR	95% CI	*p* Value
Age, y	1.028	1.001–1.056	0.039			
Sex						
Male	1.000	(ref)				
Female	1.296	0.743–2.263	0.361			
Pathology						
SCC	1.000	(ref)		1.000	(ref)	
Non-SCC	0.602	0.371–0.977	0.040	0.590	0.333–1.044	0.070
T stage						
T1–T2	1.000	(ref)				
T3–T4	0.853	0.534–1.363	0.506			
N stage						
N0	1.000	(ref)				
N1	1.473	0.600–3.611	0.398			
N2	0.710	0.329–1.532	0.383			
N3	0.740	0.359–1.526	0.415			
M stage						
M1a	1.000	(ref)				
M1b	0.924	0.516–1.653	0.789			
M1c	0.912	0.544–1.528	0.726			
Primary tumor size, cm	1.024	0.932–1.126	0.617			
PD-L1 expression						
<1	1.000	(ref)				
1–49	1.630	0.946–2.811	0.079			
≥50	1.094	0.593–2.017	0.774			
Number of metastatic lesion						
1	1.000	(ref)				
2	0.892	0.386–2.061	0.790			
3–5	0.905	0.392–2.090	0.815			
>5	0.757	0.410–1.397	0.374			
Number of metastatic organ						
1	1.000	(ref)				
2	0.489	0.266–0.901	0.022			
3	0.570	0.285–1.140	0.112			
>3	0.609	0.299–1.240	0.172			
Intracranial metastasis						
Not involved	1.000	(ref)				
Involved	0.878	0.409–1.884	0.739			
Systemic treatment scheme						
ICI	1.000	(ref)		1.000	(ref)	
ICI + CTx	0.658	0.393–1.100	0.111	0.626	0.344–1.138	0.125
ICI regimen						
PD-1	1.000	(ref)				
PD-L1	0.697	0.332–1.465	0.341			
ICI combination	1.429	0.865–2.362	0.164			

Abbreviations: HR, hazard ratio; CI, confidence interval; SCC, squamous cell carcinoma; ICI, immune checkpoint inhibitor; CTx, chemotherapy; PD-L1, programmed death ligand 1; PD-1, programmed cell death protein 1.

**Table 3:** Treatments and patterns of failure after second-line therapy in patients with disease progression.

	1 Lesion (*N* = 28)		2 Lesions (*N* = 24)		3–5 Lesions (*N* = 20)		>5 Lesions (*N* = 149)		
	*N*	%	*N*	%	*N*	%	*N*	%	*p* Value
Second-line treatment scheme *									0.130
Best supportive care	2	10.0	4	21.1	2	11.1	33	26.2	
Systemic Tx	15	75.0	11	57.9	13	72.2	83	65.9	
Local salvage Tx	1	5.0	2	10.5	0	0.0	1	0.8	
Systemic Tx + local salvage Tx	2	10.0	2	10.5	3	16.7	9	7.1	
Second-line systemic treatment *									0.070
No systemic treatment	3	15.0	6	31.6	2	11.1	34	27.0	
ICI	3	15.0	1	5.3	6	33.3	11	8.7	
CTx	10	50.0	7	36.8	8	44.4	66	52.4	
ICI + CTx	4	20.0	5	26.3	2	11.1	15	11.9	
Time to subsequent failure (months)									0.770
Median (range)	6.0 (range, 1.1–13.2)		3.8 (range, 2.0–21.1)		4.1 (range, 0.6–29.4)		4.7 (range, 0.4–49.5)		
Subsequent patterns of failure									0.043
No failure	13	46.4	11	45.8	7	35.0	84	56.4	
Previous site only	2	7.1	8	33.3	6	30.0	22	14.8	
New with/without previous site	13	46.4	5	20.8	7	35.0	43	28.9	
Limited progression at subsequent failure **									0.057
No	13	86.7	9	69.2	7	53.8	55	84.6	
Yes	2	13.3	4	30.8	6	46.2	10	15.4	

* Analyzed 20 (1 lesion), 19 (2 lesions), 18 (3–5 lesions), and 126 (>5 lesions) patients who experienced treatment failure after first-line treatment. ** Analyzed 15 (1 lesion), 13 (2 lesions), 13 (3–5 lesions), and 65 (>5 lesions) patients who experienced subsequent failure after second-line treatment. Abbreviations: ICI, immune checkpoint inhibitor; CTx, chemotherapy; Tx, therapy.

## Data Availability

Research data will be shared upon reasonable request to the corresponding author.

## Supplementary Materials

The following supporting information can be downloaded at: https://www.mdpi.com/article/10.3390/cancers18091363/s1. Figure S1: Subgroup analyses of patterns of failure according to number of metastatic lesions. (A,B) Cumulative incidence of (A) original site recurrence and (B) new site recurrence with or without original site recurrence in the PD-L1 ≥ 50% subgroup. (C,D) Cumulative incidence of (C) original site recurrence and (D) new site recurrence with or without original site recurrence in the PD-L1 < 50% subgroup. (E,F) Cumulative incidence of (E) original site recurrence and (F) new site recurrence with or without original site recurrence in the ICI monotherapy subgroup. (G,H) Cumulative incidence of (G) original site recurrence and (H) new site recurrence with or without original site recurrence in the ICI combination subgroup. (I,J) Cumulative incidence of (I) original site recurrence and (J) new site recurrence with or without original site recurrence in the SCC histology subgroup. (K,L) Cumulative incidence of (K) original site recurrence and (L) new site recurrence with or without original site recurrence in the non-SCC histology subgroup. All cumulative incidences were estimated using competing risk analysis; Figure S2: Survival outcomes according to the patterns of progression after first-line systemic therapy. (A) Overall survival according to patterns of progression. (B) Progression-free survival according to patterns of progression; Figure S3: Survival outcomes according to local salvage treatment. (A) Overall survival according to receipt of local salvage treatment. (B) Progression-free survival according to receipt of local salvage treatment; Table S1: Distribution of metastatic sites according to number of metastatic lesions; Table S2: First-line systemic therapies according to number of metastatic lesions; Table S3: Patterns of failure according to involvement of brain, liver, and bone metastases; Table S4: Patterns of initial progression and subsequent failure according to local salvage treatment.

## Abbreviations

The following abbreviations are used in this manuscript:

AP-CT	abdomino-pelvic computed tomography
CI	confidence interval
CT	computed tomography
CTx	chemotherapy
ctDNA	circulating tumor DNA
EGFR	epidermal growth factor receptor
GKS	gamma knife surgery
HR	hazard ratio
ICI	immune checkpoint inhibitor
LCT	local consolidative therapy
LN	lymph node
NSR	new site with or without original site recurrence
miRNA	microRNA
MRI	magnetic resonance imaging
NSCLC	non-small cell lung cancer
OS	overall survival
OSR	original site recurrence
PD-1	programmed cell death protein 1
PD-L1	programmed death ligand 1
PET-CT	positron emission tomography–computed tomography
PFS	progression-free survival
POF	pattern of failure
SABR	stereotactic ablative radiotherapy
SCC	squamous cell carcinoma
TKI	tyrosine kinase inhibitor
Tx	therapy
