# Supplementary material for: Patterns of Failure in Synchronous Metastatic Non-Small Cell Lung Cancer Without Driver Alterations According to Metastatic Burden
Source: Cancers (Basel). 2026 Apr 24;18(9):1363. doi: 10.3390/cancers18091363 (PMC13162817; doi:10.3390/cancers18091363)
Supplement: Supplementary file 1 [file cancers-18-01363-s001.zip › cancers-4260601-supplementary.pdf]

Supplementary Figure S1. Subgroup analyses of patterns of failure according to number of metastatic lesions

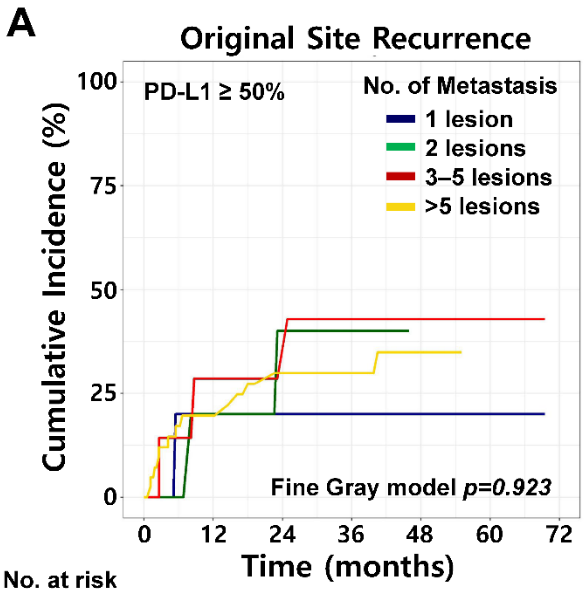

No. at risk	0	12	24	36	48	60	72
1 lesion	5	4	3	2	1	1	0
2 lesions	5	4	3	2	0	0	0
3–5 lesions	7	2	2	1	1	1	1
>5 lesions	42	17	9	7	2	0	0

Group	HR	95% CI	*p* value
1 lesion	1	(ref)	
2 lesions	1.922	0.186–19.903	0.580
3–5 lesions	2.184	0.230–20.707	0.500
>5 lesions	1.675	0.212–13.232	0.620

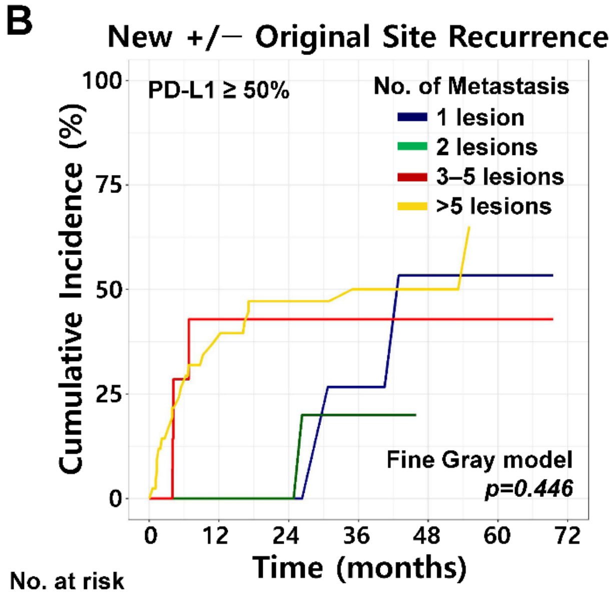

No. at risk	0	12	24	36	48	60	72
1 lesion	5	4	3	2	1	1	0
2 lesions	5	4	3	2	0	0	0
3–5 lesions	7	2	2	1	1	1	1
>5 lesions	42	17	9	7	2	0	0

Group	HR	95% CI	*p* value
1 lesion	1	(ref)	
2 lesions	0.506	0.062–4.160	0.530
3–5 lesions	1.357	0.268–6.875	0.710
>5 lesions	1.807	0.562–5.811	0.320

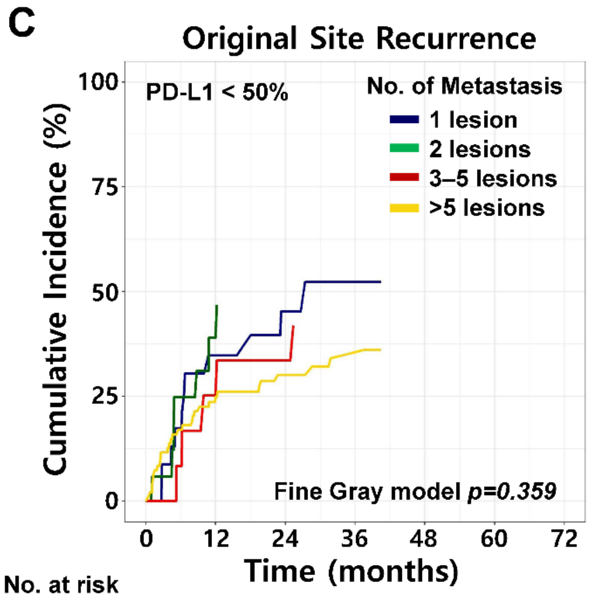

No. at risk	0	12	24	36	48	60	72
1 lesion	23	10	5	2	1	1	1
2 lesions	17	1	0	0	0	0	0
3–5 lesions	13	3	1	0	0	0	0
>5 lesions	95	24	7	4	1	1	1

Group	HR	95% CI	*p* value
1 lesion	1	(ref)	
2 lesions	0.963	0.383–2.422	0.940
3–5 lesions	0.771	0.292–2.035	0.600
>5 lesions	0.643	0.331–1.249	0.190

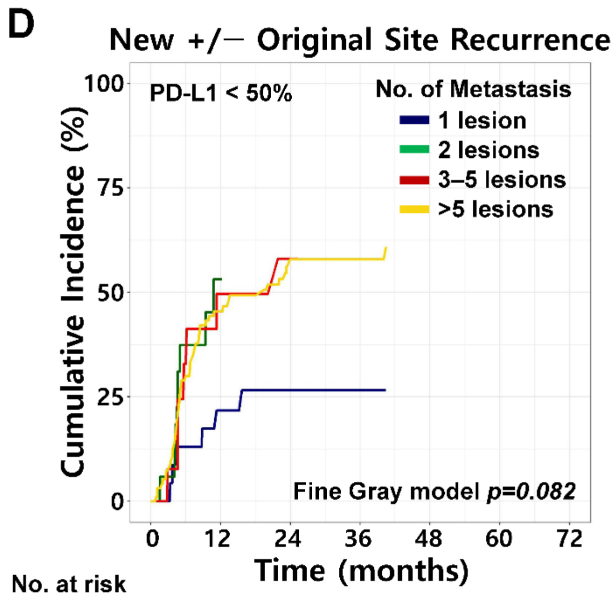

No. at risk	0	12	24	36	48	60	72
1 lesion	23	10	5	2	1	1	1
2 lesions	17	1	0	0	0	0	0
3–5 lesions	13	3	1	0	0	0	0
>5 lesions	95	24	7	4	1	1	1

Group	HR	95% CI	*p* value
1 lesion	1	(ref)	
2 lesions	2.502	0.843–7.425	0.099
3–5 lesions	2.680	0.910–7.896	0.074
>5 lesions	2.761	1.180–6.461	0.019

E

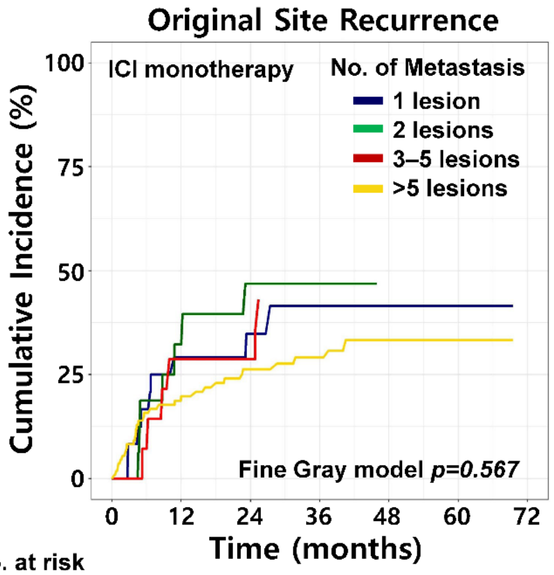

No. at risk							
1 lesion	24	12	7	3	2	2	1
2 lesions	16	5	3	2	0	0	0
3–5 lesions	15	3	2	0	0	0	0
>5 lesions	110	37	15	10	3	2	2

Group	HR	95% CI	*p* value
1 lesion	1	(ref)	
2 lesions	1.181	0.453–3.076	0.730
3–5 lesions	1.056	0.401–2.783	0.910
>5 lesions	0.739	0.355–1.541	0.420

F

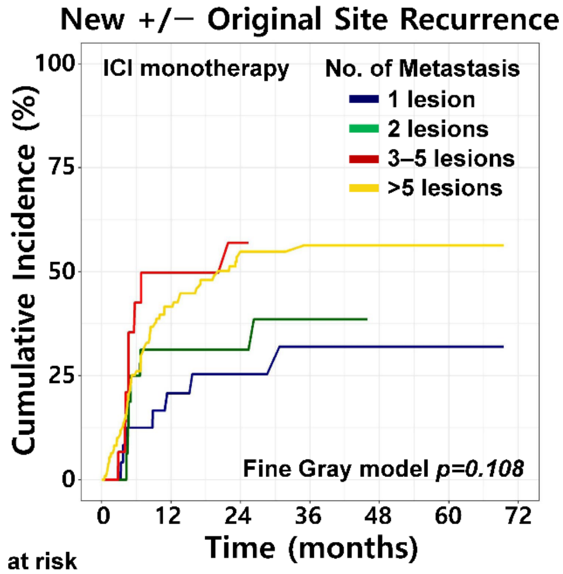

No. at risk							
1 lesion	24	12	7	3	2	2	1
2 lesions	16	5	3	2	0	0	0
3–5 lesions	15	3	2	0	0	0	0
>5 lesions	110	37	15	10	3	2	2

Group	HR	95% CI	*p* value
1 lesion	1	(ref)	
2 lesions	1.403	0.474–4.154	0.540
3–5 lesions	2.514	0.897–7.043	0.080
>5 lesions	2.291	1.060–4.950	0.035

G

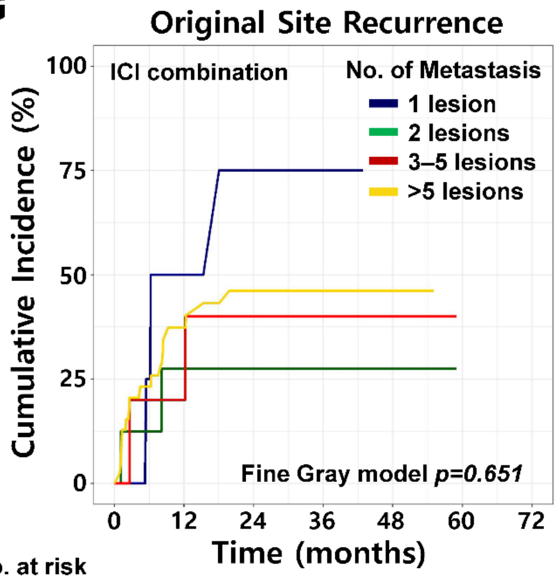

No. at risk							
1 lesion	4	2	1	1	0	0	0
2 lesions	8	1	1	1	1	0	0
3–5 lesions	5	2	1	1	1	1	1
>5 lesions	39	7	3	2	1	0	0

Group	HR	95% CI	*p* value
1 lesion	1	(ref)	
2 lesions	0.320	0.060–1.705	0.180
3–5 lesions	0.455	0.095–2.194	0.330
>5 lesions	0.569	0.212–1.524	0.260

H

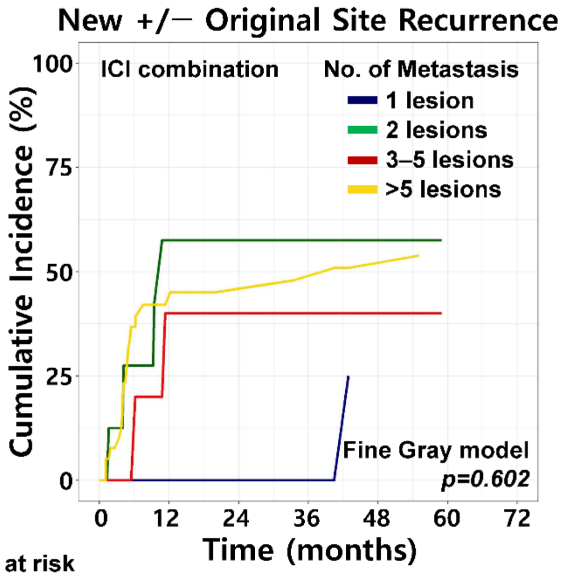

No. at risk							
1 lesion	4	2	1	1	0	0	0
2 lesions	8	1	1	1	1	0	0
3–5 lesions	5	2	1	1	1	1	1
>5 lesions	39	7	3	2	1	0	0

Group	HR	95% CI	*p* value
1 lesion	1	(ref)	
2 lesions	3.302	0.473–23.042	0.230
3–5 lesions	1.850	0.225–15.214	0.570
>5 lesions	3.063	0.535–17.544	0.210

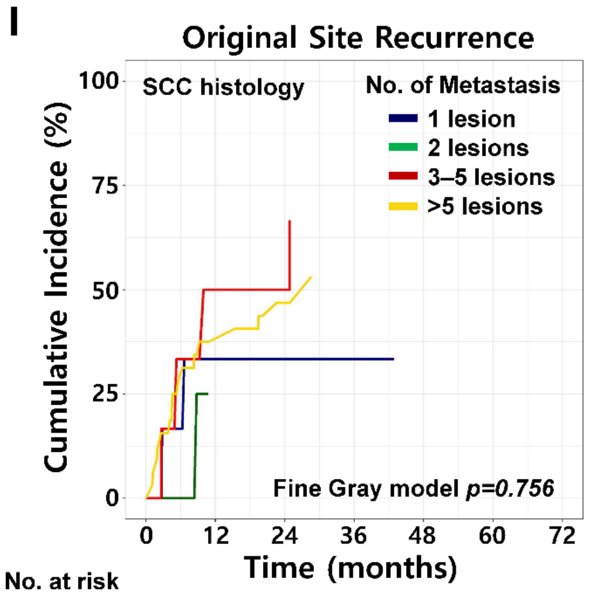

No. at risk							
1 lesion	6	2	1	1	0	0	0
2 lesions	4	0	0	0	0	0	0
3–5 lesions	6	1	1	0	0	0	0
>5 lesions	32	8	2	0	0	0	0

Group	HR	95% CI	*p* value
1 lesion	1	(ref)	
2 lesions	0.637	0.062–6.588	0.700
3–5 lesions	2.147	0.418–11.028	0.360
>5 lesions	1.595	0.359–7.056	0.540

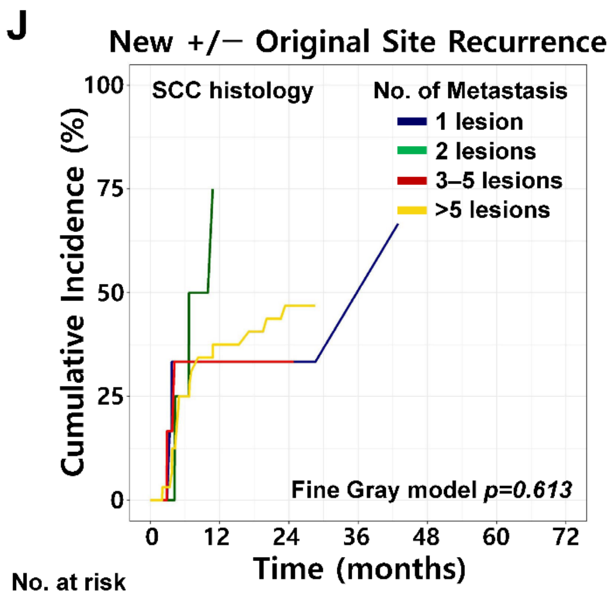

No. at risk							
1 lesion	6	2	1	1	0	0	0
2 lesions	4	0	0	0	0	0	0
3–5 lesions	6	1	1	0	0	0	0
>5 lesions	32	8	2	0	0	0	0

Group	HR	95% CI	*p* value
1 lesion	1	(ref)	
2 lesions	1.624	0.364–7.246	0.520
3–5 lesions	0.628	0.090–4.365	0.640
>5 lesions	0.840	0.242–2.916	0.780

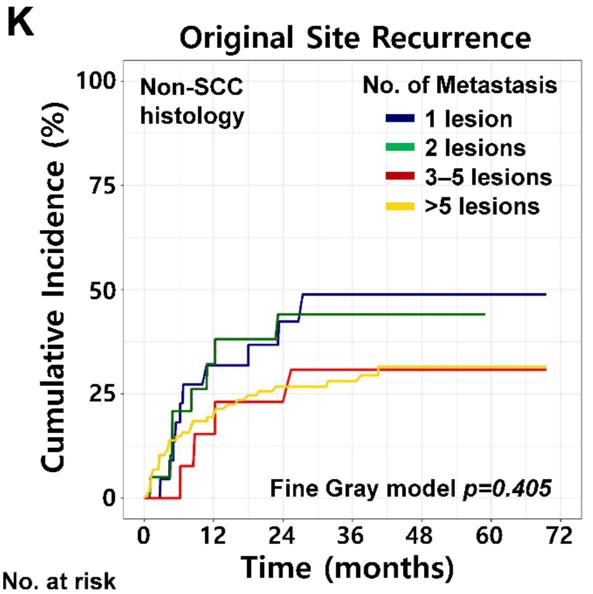

No. at risk							
1 lesion	22	12	7	3	2	2	1
2 lesions	20	6	4	3	1	0	0
3–5 lesions	14	4	2	1	1	1	1
>5 lesions	117	36	16	12	4	2	2

Group	HR	95% CI	*p* value
1 lesion	1	(ref)	
2 lesions	0.940	0.384–2.301	0.890
3–5 lesions	0.576	0.195–1.698	0.320
>5 lesions	0.597	0.303–1.179	0.140

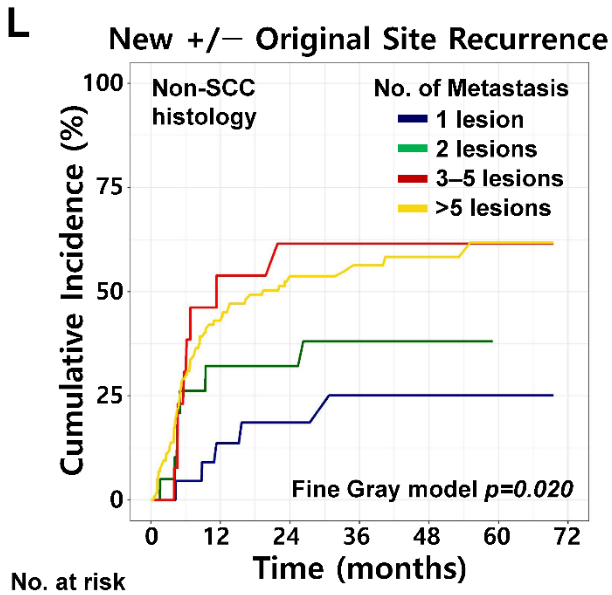

No. at risk							
1 lesion	22	12	7	3	2	2	1
2 lesions	20	6	4	3	1	0	0
3–5 lesions	14	4	2	1	1	1	1
>5 lesions	117	36	16	12	4	2	2

Group	HR	95% CI	*p* value
1 lesion	1	(ref)	
2 lesions	1.919	0.620–5.946	0.260
3–5 lesions	3.529	1.210–10.293	0.021
>5 lesions	3.390	1.422–8.080	0.006

Supplementary Figure S1. Subgroup analyses of patterns of failure according to number of metastatic lesions. (A, B) Cumulative incidence of (A) original site recurrence and (B) new site recurrence with or without original site recurrence in the PD-L1 ≥ 50% subgroup. (C, D) Cumulative incidence of (C) original site recurrence and (D) new site recurrence with or without original site recurrence in the PD-L1 < 50% subgroup. (E, F) Cumulative incidence of (E) original site recurrence and (F) new site recurrence with or without original site recurrence in the ICI monotherapy

subgroup. (G, H) Cumulative incidence of (G) original site recurrence and (H) new site recurrence with or without original site recurrence in the ICI combination subgroup. (I, J) Cumulative incidence of (I) original site recurrence and (J) new site recurrence with or without original site recurrence in the SCC histology subgroup. (K, L) Cumulative incidence of (K) original site recurrence and (L) new site recurrence with or without original site recurrence in the non-SCC histology subgroup. All cumulative incidences were estimated using competing risk analysis.

Supplementary Figure S2. Survival outcomes according to the patterns of progression after first-line systemic therapy

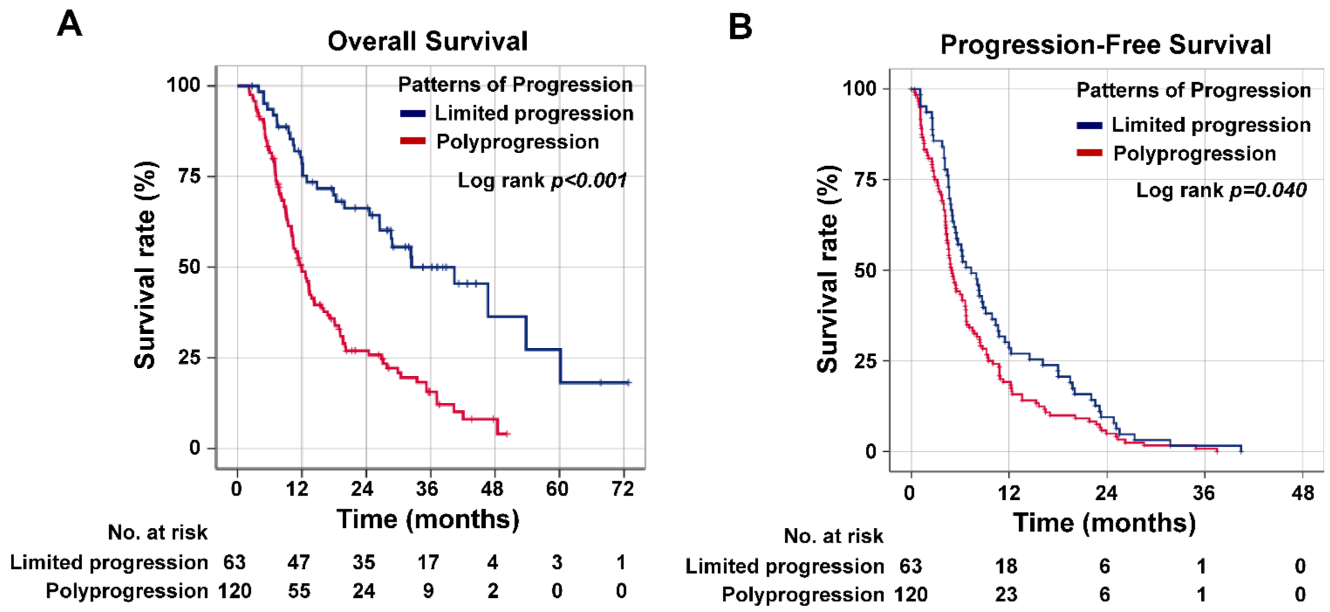

Supplementary Figure S2. Survival outcomes according to patterns of progression after first-line systemic therapy. (A) Overall survival according to patterns of progression. (B) Progression-free survival according to patterns of progression.

Supplementary Figure S3. Survival outcomes according to local salvage treatment

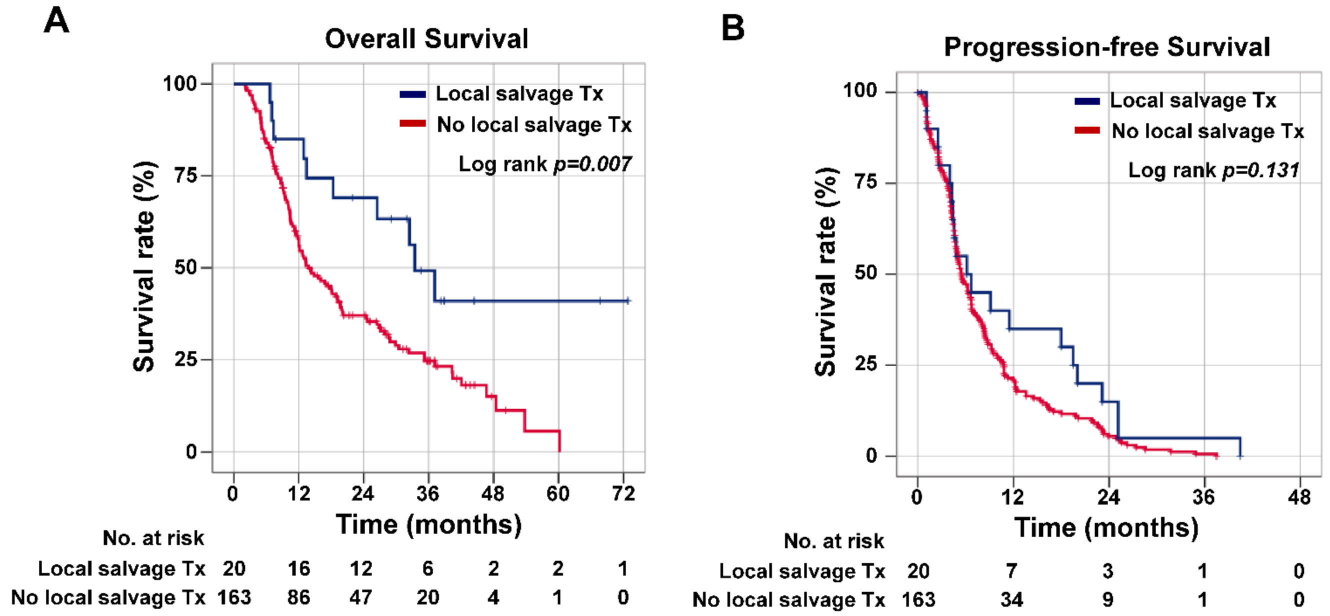

Supplementary Figure S3. Survival outcomes according to local salvage treatment. (A) Overall survival according to receipt of local salvage treatment. (B) Progression-free survival according to receipt of local salvage treatment.

Supplementary Table S1. Distribution of metastatic sites according to number of metastatic lesions

	1 lesion (N=28)		2 lesions (N=24)		3–5 lesions (N=20)		>5 lesions (N=149)		
	N	%	N	%	N	%	N	%	P value
**Contralateral lung**									0.009
**Uninvolved**	23	82.1	22	91.7	13	65.0	92	61.7	
**Involved**	5	17.9	2	8.3	7	35.0	57	38.3	
**Pleural seeding**									<0.001
**Uninvolved**	28	100.0	24	100.0	20	100.0	93	62.4	
**Involved**	0	0.0	0	0.0	0	0.0	56	37.6	
**Pleural effusion**									<0.001
**Uninvolved**	28	100.0	24	100.0	20	100.0	68	45.6	
**Involved**	0	0.0	0	0.0	0	0.0	81	54.4	
**Pericardial effusion**									0.065
**Uninvolved**	28	100.0	24	100.0	20	100.0	135	90.6	
**Involved**	0	0.0	0	0.0	0	0.0	14	9.4	
**Lymphangitic metastasis**									0.260
**Uninvolved**	28	100.0	24	100.0	20	100.0	141	94.6	
**Involved**	0	0.0	0	0.0	0	0.0	8	5.4	
**Distant LN**									0.403
**Uninvolved**	21	75.0	15	62.5	20	100.0	106	71.1	
**Involved**	7	25.0	9	37.5	0	0.0	43	28.9	
**Chestwall**									0.571
**Uninvolved**	28	100.0	23	95.8	20	100.0	143	96.0	
**Involved**	0	0.0	1	4.2	0	0.0	6	4.0	
**Brain**									0.479
**Uninvolved**	24	85.7	21	87.5	17	85.0	138	92.6	
**Involved**	4	14.3	3	12.5	3	15.0	11	7.4	
**Bone**									0.064
**Uninvolved**	22	78.6	19	79.2	17	85.0	91	61.1	
**Involved**	6	21.4	5	20.8	3	15.0	58	38.9	
**Liver**									0.284
**Uninvolved**	28	100.0	22	91.7	18	90.0	135	90.6	
**Involved**	0	0.0	2	8.3	2	10.0	14	9.4	
**Adrenal gland**									0.008
**Uninvolved**	24	85.7	15	62.5	10	50.0	132	88.6	
**Involved**	4	14.3	9	37.5	10	50.0	17	11.4	
**Pancreas**									0.557
**Uninvolved**	28	100.0	23	95.8	20	100.0	147	98.7	
**Involved**	0	0.0	1	4.2	0	0.0	2	1.3	

**Kidney**									0.299
**Uninvolved**	28	100.0	22	91.7	20	100.0	144	96.6	
**Involved**	0	0.0	2	8.3	0	0.0	5	3.4	
**Spleen**									0.337
**Uninvolved**	28	100.0	23	95.8	20	100.0	148	99.3	
**Involved**	0	0.0	1	4.2	0	0.0	1	0.7	
**Muscle/soft tissue**									0.575
**Uninvolved**	26	92.9	24	100.0	19	95.0	138	92.6	
**Involved**	2	7.1	0	0.0	1	5.0	11	7.4	
**Peritoneal carcinomatosis**									0.579
**Uninvolved**	28	100.0	24	100.0	20	100.0	145	97.3	
**Involved**	0	0.0	0	0.0	0	0.0	4	2.7	

Supplementary Table S2. First-line systemic therapies according to number of metastatic lesions.

	1 lesion (N=28)		2 lesions (N=24)		3–5 lesions (N=20)		>5 lesions (N=149)		
	N	%	N	%	N	%	N	%	P value
**Systemic treatment scheme**									0.888
**ICI**	6	21.4	4	16.7	4	20.0	35	23.5	
**ICI + CTx**	22	78.6	20	83.3	16	80.0	114	76.5	
**ICI regimen**									0.787
**PD-1**	19	67.9	14	58.3	12	60.0	88	59.1	
**PD-L1**	5	17.9	2	8.3	3	15.0	22	14.8	
**ICI combination**	4	14.3	8	33.3	5	25.0	39	26.2	

Abbreviations: ICI, immune checkpoint inhibitor; CTx, chemotherapy; PD-L1, programmed death ligand 1; PD-1, programmed cell death protein 1.

Supplementary Table S3. Patterns of failure according to involvement of brain, liver, and bone metastases

	Brain			Liver			Bone		
	Uninvolved N (%)	Involved N (%)	P value	Uninvolved N (%)	Involved N (%)	P value	Uninvolved N (%)	Involved N (%)	P value
**All patient group**			0.227			0.259			0.009
**No failure**	37 (18.5)	1 (4.8)		37 (18.3)	1 (5.3)		31 (21.8)	7 (8.9)	
**Original site only**	69 (34.5)	7 (33.3)		70 (34.7)	6 (31.6)		52 (36.6)	24 (30.4)	
**New with/without original site**	94 (47.0)	13 (61.9)		95 (47.0)	12 (63.2)		59 (41.5)	48 (60.8)	
**Patient with 1 metastatic lesion**			0.380			NA			0.300
**No failure**	8 (33.3)	0 (0)		8 (28.6)	0 (0)		7 (31.8)	1 (16.7)	
**Original site only**	9 (37.5)	2 (50.0)		11 (39.3)	0 (0)		7 (31.8)	4 (66.7)	
**New with/without original site**	7 (29.2)	2 (50.0)		9 (32.1)	0 (0)		8 (36.4)	1 (16.7)	
**Patient with 2 metastatic lesions**			0.537			0.217			0.337
**No failure**	5 (23.8)	0 (0)		5 (22.7)	0 (0)		5 (26.3)	0 (0)	
**Original site only**	8 (38.1)	1 (33.3)		9 (40.9)	0 (0)		6 (31.6)	3 (60.0)	
**New with/without original site**	8 (38.1)	2 (66.7)		8 (36.4)	2 (100.0)		8 (42.1)	2 (40.0)	
**Patient with 3‒5 metastatic lesions**			0.171			0.071			0.111
**No failure**	2 (11.8)	0 (0)		2 (11.8)	0 (0)		0 (0)	2 (20.0)	
**Original site only**	8 (47.1)	0 (0)		5 (29.4)	3 (100.0)		6 (60.0)	2 (20.0)	
**New with/without original site**	7 (41.2)	3 (100.0)		10 (58.8)	0 (0)		4 (40.0)	6 (60.0)	
**Patient with >5 metastatic lesions**			0.825			0.313			0.008
**No failure**	22 (15.9)	1 (9.1)		22 (16.3)	1 (7.1)		19 (20.9)	4 (6.9)	
**Original site only**	44 (31.9)	4 (36.4)		45 (33.3)	3 (21.4)		33 (36.3)	15 (25.9)	
**New with/without original site**	72 (52.2)	6 (54.5)		68 (50.4)	10 (71.4)		39 (42.9)	39 (67.2)	

Supplementary Table S4. Patterns of initial progression and subsequent failure according to local salvage treatment

	No local salvage treatment (N=163)		Local salvage treatment (N=20)		
	**N**	**%**	**N**	**%**	**P value**
**Patterns of initial progression***					<0.001
**Polyprogression**	116	71.2	4	20.0	
**Limited progression**	47	28.8	16	80.0	
**Subsequent patterns of failure**					0.022
**No failure**	73	44.8	4	20.0	
**Previous site only**	35	21.5	3	15.0	
**New with/without previous site**	55	33.7	13	65.0	

***Analyzed 163 (no local salvage treatment) and 20 (local salvage treatment) patients who experienced failure after first-line treatment.**
